# Enhanced isolation of lymphoid cells from human skin

**DOI:** 10.1111/ced.12802

**Published:** 2016-01-25

**Authors:** M. Salimi, S. Subramaniam, T. Selvakumar, X. Wang, S. Zemenides, D. Johnson, G. Ogg

**Affiliations:** ^1^ MRC Human Immunology UnitNIHR Biomedical Research CentreRadcliffe Department of MedicineUniversity of OxfordOxfordUK; ^2^ Department of Periodontology and Oral MedicineSchool of StomatologyFourth Military Medical UniversityXi'anShaanxiChina; ^3^ Department of Plastic and Reconstructive SurgeryJohn Radcliffe HospitalOxford University Hospitals NHS TrustOxfordUK

## Abstract

Studying skin immune cells under various pathophysiological conditions is vital for understanding the nature of cutaneous inflammatory responses. Available methods of isolating cells from the skin have relatively low yield or require *in vitro* culture. To increase the effective isolation of skin immune cells, we used collagenase P treatment. The number of T cells obtained *ex vivo* using this technique was dramatically greater than that obtained with conventional methods, without the need for long‐term culture. The phenotype and function of isolated cells were comparable with those of cells isolated by EDTA treatment. Collagenase P‐based methods will enhance the ability to investigate lymphoid cell function in both healthy and diseased skin.

A diverse repertoire of T cells and B cells resides in the skin, and indeed, it has been estimated that the number of resident T cells in normal human skin is almost 2 × 10^10^, which is nearly double the number of T cells in the circulating blood.^1^ Established methods using EDTA or collagenase D produce a low yield of cells^1^, ^2^ and so other approaches have introduced a culture step, for example on the surface of Cellfoam three‐dimensional growth matrices for 21 days.^3^ Although such culture approaches represent a significant step forward for certain applications, they can introduce potential *in vitro* changes in the frequency of the isolated cells. Effective isolation of lymphoid cells from the skin is vital for maximizing information obtained from skin samples in order to define their role in health and disease. It is clear that T cells play a role in diverse skin surveillance and pathology, and understanding their role may contribute to the development of novel therapeutic approaches. We report a new method that gives rise to a far larger number of intact T cells *ex vivo* from human skin than has previously been possible.

## Report

T cells were isolated from samples of normal healthy adult skin using EDTA (*n* = 4) and collagenase D digestion (*n* = 4), as described previously^2^, ^4^ and in supplementary methods online. In addition, we used an alternative approach in which skin biopsies were first washed with cold phosphate‐buffered saline, and the subcutaneous fat was removed. The biopsies were cut into pieces < 0.5 mm in size, placed in RPMI 1640 (Sigma‐Aldrich, Poole, Dorset, UK) supplemented with 10% heat‐inactivated fetal calf serum (FCS), 2 mmol/L l‐glutamine, 100 IU/mL penicillin, 100 μg/mL streptomycin and 1 mg/mL collagenase P (cat. no. 11213865001; Roche, Burgess Hill, West Sussex, UK), and incubated overnight at 37 °C. With the use of a pipette, the mixture was then repeatedly sucked up and expelled to homogenize the tissue further. To reduce free DNA fragments, endonuclease deoxyribonuclease (DNase) I was added at 200 Kunitz units/mL (cat. no. 10104159001; Roche) for 15 min at room temperature. The tissue was passed through a 100 μm nylon mesh strainer, then through a 70 μm nylon mesh strainer (734‐0004 and 734‐0003; VWR, Lutterworth, Leicestershire, UK) and washed with ice‐cold 10 mmol/L EDTA solution (10× the volume of collagenase solution). After the solution was spun at 300 ***g*** for 20 min at 4 °C, the cell pellet was resuspended in cold RPMI medium containing 10% FCS and passed through a 40 μm tissue strainer (734‐0002; VWR). Larger samples (<50 mm diameter) were further purified using Ficoll density gradient purification.

**Figure 1:**
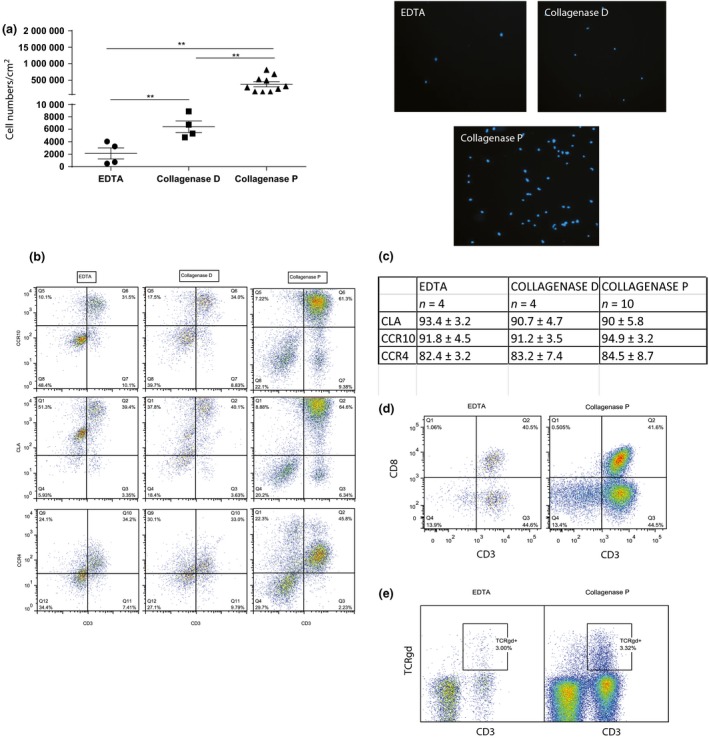
Collagenase P enzymatic treatment dramatically increases skin lymphoid cell isolation yield with intact expression of CD3 and CD8. (a) Skin samples were cut into small pieces and treated with EDTA, collagenase D or collagenase P. Frequencies of isolated cells were measured using 0.4% Trypan blue exclusion. EDTA treatment and collagenase D treatment resulted in the isolation of 2130 ± 889 and 6417 ± 927 cells/cm^2^ respectively, while collagenase P treatment increased the number of isolated cells to 303 234 ± 68 321 cells/cm^2^. Hoechst staining of isolated cells using different protocols is shown on the right. (b) Expression of skin homing markers CLA, CCR10 and CCR4 were compared on lymphoid cells using the different methods of cell isolation. (c) Mean frequencies on CD45+ CD3+ cells are summarized. (d) Live CD45+ cells isolated by EDTA and collagenase P treatment were stained for CD3 and CD8 expression. Similar frequencies of CD8+ and CD8− cells were observed using the different methods. (e) Frequencies of CD3+ γδ T cells isolated by collagenase P treatment were similar to the EDTA method. **P < 0.05.

The cells were then counted using 0.4% Trypan blue exclusion. On average, EDTA treatment and collagenase D treatment respectively resulted in the isolation of 2130 ± 889 and 6417 ± 927 cells/cm^2^ from the skin biopsies, while the new approach based on collagenase P treatment (*n* = 10) dramatically increased the number of isolated cells to 303 234 ± 68 321 cells/cm^2^ (Fig. 1a; Hoechst staining of isolated cells using different protocols is shown on the right).

Depending on the first encounter of naive T cells with antigen, activated effector memory cells are imprinted with preferential homing markers. Selective imprinting of T cells is essential for effective recruitment and robust immune responses in tissues, and is influenced by the microenvironment and professional antigen‐presenting cells. To evaluate the phenotype of T cells isolated using collagenase P treatment, we compared the expression of the skin homing markers cutaneous lymphocyte antigen (CLA)^5^ and chemokine receptors CCR4 and CCR10. We found that > 90% of T cells isolated from the skin expressed CLA and CCR10, while > 70% expressed CCR4. Collagenase P did not alter the expression of homing markers on T cells isolated from skin (Fig. 1b,c).

To ensure that our method did not alter the cell surface expression of CD3, TCR and CD8 markers compared with EDTA or collagenase D, we investigated expression of markers on skin‐resident T cells.^6^, ^7^ CD3 and CD8 were expressed at similar proportions in the cells isolated by different methods (Fig. 1d).

γδ T cells are another important subset of T cells that are believed to contribute to psoriasis skin inflammation. They are known to be expressed in small numbers in human skin, and so we examined whether the new method could detect such a minor population. The percentage of γδ T cells (3.63 ± 0.77% of live cells) detected following collagenase P treatment of healthy skin donors was also similar to earlier reports that used other methods of skin T cell isolation (Fig. 1e).^8^, ^9^

**Figure 2:**
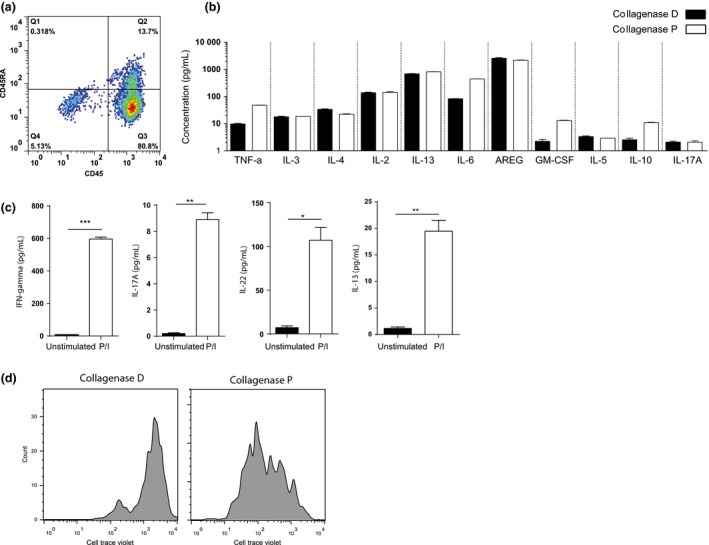
Skin‐resident T cells had a memory phenotype and were functionally intact. (a) Less than 10% of T cells isolated by the new method of collagenase P treatment showed a naive phenotype and expressed CD45RA. (b) Multiplex cytokine analysis of *ex vivo* phorbol myristate acetate (PMA)/ionomycin‐activated total skin cells isolated by collagenase P and collagenase D treatments. (c) Expression of interferon‐γ, interleukin (IL)‐13, IL‐17A and IL‐22 by skin‐resident T cells isolated by collagenase P as measured by ELISA after stimulation with PMA/ionomycin. (d) Proliferative capacity of CellTrace Violet‐labelled T cells isolated by collagenase D and collagenase P treatment following 5 days of culture with IL‐2. *P < 0.02, **P < 0.002, ***P < 0.0002.

Further examination of cell surface phenotype showed that most of the skin‐resident T cells had a memory phenotype (Fig. 2a). We next compared the ability of total skin cells isolated by collagenase D and collagenase P methods in producing different cytokines after stimulation with phorbol myristate acetate and ionomycin for 4 h by multiplex cytokine array. Cells isolated using collagenase P treatment produced similar or higher amounts of cytokines compared with collagenase D treatment (Fig. 2b). Investigation of different subpopulations of T cells in the skin for their functional integrity after collagenase P digestion identified production of interferon γ, interleukin (IL)‐17, IL‐22 and IL‐13 by activated T cells as determined by ELISA (Fig. 2c). Less IL‐13 was found, suggesting that non‐T cells represent a source of IL‐13 in the skin. To further demonstrate the efficacy of collagenase P treatment in isolating functionally active T cells, we tested the proliferative capacity of these cells. Greater numbers of cell divisions were observed in CellTrace Violet‐labelled T cells isolated by collagenase P treatment following 5 days of culture with IL‐2 (Fig. 2d).

These investigations demonstrate that the cells isolated *ex vivo* from human skin show rapid effector function and that cells producing different cytokines can be identified.

Effective isolation of lymphoid cells from human skin *ex vivo* is vital for understanding their role in health and disease. Alternative approaches have been based on cell culture steps, which have been very useful additions to possible methodologies, but require a degree of experience and laboratory infrastructure, and the culture step may introduce potential artefacts.^3^ In the current study, we have defined a new method of isolating large number of cells from skin tissue. Using a collagenase P enzymatic treatment step rather than conventional collagenase D digestion yielded far more cells with intact phenotype and function.^1^ Collagenase P is a metalloproteinase that cleaves collagen into smaller peptide fragments.^10^ It is secreted by *Clostridium histolyticum*, and has 10‐fold higher collagenase activity than collagenase D as well as higher tryptic activity. It has been used to isolate pancreatic islet cells,^11^, ^12^, ^13^ human mesenchymal stem cells,^14^ and myocardial fibroblasts.^15^

T cells are thought to play a role in the pathogenesis of many common inflammatory skin conditions such as atopic dermatitis, psoriasis and contact dermatitis. Inhibiting T‐cell function may contribute to the activity of therapeutic intervention. We have developed a novel approach to isolate lymphoid cells from human skin that greatly increases the number of viable cells obtained *ex vivo*. We anticipate that the method will be of value to those studying lymphoid populations of skin‐resident cells including T cells or innate lymphoid cells in small patient samples.^16^

**Learning points**

- Collagenase P treatment isolated a far larger number of T cells *ex vivo* from human skin than has previously been possible.
- The treatment did not modify phenotype or function of isolated T cells from skin biopsies.
- It also did not alter the expression of homing markers on T cells isolated from the skin.
- The percentage of γδ T cells (3.63 ± 0.77% of live cells) detected following collagenase P treatment of healthy skin donors was similar to those in earlier reports that used other methods of skin T‐cell isolation.
- The cells isolated *ex vivo* from human skin by collagenase P digestion produce cytokines.
- T cells isolated by collagenase P treatment show high proliferative capacity.

## Supporting information


**Data S1.** Supplementary material and methods.
