# Supplementary material for: Enhanced isolation of lymphoid cells from human skin
Source: Clin Exp Dermatol. 2016 Jan 25;41(5):552–6. doi: 10.1111/ced.12802 (PMC4981906; doi:10.1111/ced.12802)
Supplement: Supplementary file 1 — Data S1. Supplementary material and methods. [file CED-41-552-s001.docx]

# Supplementary material and methods

## Skin tissue

Normal adult human skin surplus tissue was obtained from surgical procedures according to GCP guidance with ethics approval from the Oxford Research Ethics Committee.

## Isolating immune cells using established methods

After removing the subcutaneous fat, skin biopsies were cut into small pieces (0.5 × 0.5 mm) and incubated in 5 mmol/L EDTA/Hank balanced salt solution with vigorous shaking at 4 °C. After 2 h, the supernatants were spun down and the skin fragments were crushed through a 40 μm strainer and pooled to obtain the maximum number of cells.^2^ For collagenase D digestion, skin biopsies were prepared as described above and incubated in RPMI 1640 medium containing 1 mg/mL collagenase D on a shaker at 37 °C. After 30 min, the skin fragments were washed with cold 10 mmol/L EDTA to stop digestion. The remaining tissue was homogenized through 40 μm nylon mesh and spun down.^2,4^

## Flow cytometry studies of isolated skin T cells

Isolated T cells were analysed using antihuman CD3 (SK7; BD biosciences) and a combination of antibodies specific to skin homing receptors: cutaneous lymphocyte associated antigen CLA (HECA-4520), CCR4 (TG6/CCR4)) and CCR10 (6588) (all Biolegend). To compare memory and naive populations, T cells were stained with anti-CD45RA (HI100) and anti-CD45RO (UCHL1) (both BD Biosciences). Monoclonal antibodies against γδ T-cell receptor (B1.1) was obtained from eBiosciences. The samples were acquired using FACSDiva or Summit software on an LSRFortessa or CyAn flow cytometer, respectively. FlowJo and Summit software were used for further data analysis.

## ELISA for interferon-γ and interleukins 13, 17 and 22

ELISA for interferon-γ, interleukin (IL)-13, IL-22 and IL-17 (cat. nos 88-7316-88, 88-7439-86, 88-7522 and 88-7176 respectively; eBiosciences) were performed according to the manufacturer’s instructions in the presence or absence of PMA/ionomycin overnight stimulation. Briefly, ELISA plats (Coat Corning Costar 9018; Nunc Maxisorp^®^) were coated with 100 μL/well capture antibody and incubated overnight at 4 °C. After 3 washes the plate was blocked for 1 h with assay buffer and 100 μL of supernatant were added to each well and incubated for 2 h in room temperature. After five washes, samples were incubated with biotinylated detection antibody for 1 h before streptavidin–horseradish peroxidase incubation for further 30 min, then 100 μL substrate solution were added to each well for 10 min, after which the reaction was stopped and the plates read at 450 nm wavelength.

## Statistical analysis

Statistical analysis was carried out using *t*-tests performed with GraphPad Prism (v6.00; GraphPad Software, San Diego, USA).
